# Supplementary material for: Novel strategy for wide-range wind vector measurement using the hybrid CP/CTD heating mode and sequential measuring and correcting
Source: PLoS One. 2021 Jul 8;16(7):e0254256. doi: 10.1371/journal.pone.0254256 (PMC8266126; doi:10.1371/journal.pone.0254256)
Supplement: S1 Nomenclature — (DOCX) [file pone.0254256.s002.docx]

**Nomenclature**

*A*, *B* parameters related to the environment surrounding the system

*α* temperature coefficient

*β* amplification

CP constant power

CTD constant temperature difference

*D_S_*[*θ_n_*] wind velocity increment

*dT* temperature variation of the heating element

*E* predefined minimum value

*e*(*k*) difference between the actual and target compensation voltages

*I* current

*K_d_* differential coefficient

*K_i_* integral coefficient

*K_p_* proportional coefficient

*P* power of the heating component

PCB printed circuit board

PID proportional–integral–derivative

*R* standard resistance of the PT100 thermistor at 0 °C

*R_T_* resistance of the PT100 thermistor at temperature *T*

*T* measured temperature of the coil

*T_A_* ambient temperature

*T_0_* critical temperature of the coil

*T_S_* coil temperature

*θ* angle between the airflow tunnel and wind direction

*θ_H_* position at which the airflow tunnel is parallel to the external wind vector

*θ'_H_* corrected value of *θ_H_*

*θ_V_* position at which the airflow tunnel is perpendicular to the external wind vector

*U_A_* voltage output at the ambient temperature

*U_C_*(*k*) controller output voltage for heating power compensation

*U_d_* driving voltage

*U_S_* voltage signal output from the analog front-end circuit after the temperature sensor

*U_0_* preset heating voltage of the coil in CP mode

UV ultraviolet

*V* wind velocity

*V_S_* wind velocity output by any sensor

*V'_S_* first derivative of *V_S_*

*V''_S_* second derivative of *V_S_*

*V_S1_* output of sensor 1

*V_S2_* output of sensor 2

*V_S3_* output of sensor 3

*V_S_*[*θ_n_*] *n*^th^ sample in the sequential samples of the wind velocity output following the rotation angle
