# Supplementary material for: Novel strategy for wide-range wind vector measurement using the hybrid CP/CTD heating mode and sequential measuring and correcting
Source: PLoS One. 2021 Jul 8;16(7):e0254256. doi: 10.1371/journal.pone.0254256 (PMC8266126; doi:10.1371/journal.pone.0254256)
Supplement: Attachment — Submitted filename: rebuttal letter.docx [file pone.0254256.s003.docx]

Mehrdad Ahmadi Kamarposhti

Academic Editor

*PLOS ONE*

Dear Dr. Kamarposhti,

We would like to resubmit the attached manuscript entitled “**Novel strategy for wide-range wind vector measurement using the hybrid CP/CTD heating mode and sequential measuring and correcting.**” The manuscript ID is **PONE-D-21-04271**.

The manuscript has been carefully rechecked and appropriate changes have been made in accordance with the reviewers’ suggestions. We have also carefully proof-read the manuscript to minimize typographical, grammatical, and bibliographical errors. The responses to the reviewers’ comments have also been prepared and are attached herewith.

We thank you and the reviewers for your thoughtful suggestions and insights, which have enriched the manuscript and produced a more balanced and better account of the research. We hope that the revised manuscript is now suitable for publication in *PLOS ONE*.

Thank you for your consideration. I look forward to hearing from you.

Yours sincerely,

Xiaoyu Yu

School of Atmospheric Sciences,

Sun Yat-sen University, Zhuhai 519000, China

Email: sysyuxiaoyu@163.com

**Response to Reviewer #1’s Comments:**

Thank you for your kind comments on this paper!

Regarding the English writing issues, we have consulted a 3rd-party proofreading service provider for full-text checking on grammar and spelling. Many issues have been found and corrected now.

Regarding the nomenclature section, we have added in the manuscript, and explained all variables in every formula.

Regarding the references, we added more supplemental references and re-arranged in the text. Now a total of 42 papers are referenced.

Regarding the figures, we tweaked the description texts in the manuscript and the resolution have also been tweaked for better showing.

Regarding the future work and potential limitations, we have talked in the last paragraph of the Conclusion part.
